# Starvation induces changes in abundance and small RNA cargo of extracellular vesicles released from *Plasmodium falciparum* infected red blood cells

**DOI:** 10.1038/s41598-023-45590-6

**Published:** 2023-10-27

**Authors:** Leonie Vetter, Amanj Bajalan, Mohammad Tanvir Ahamed, Caterina Scasso, Sulman Shafeeq, Björn Andersson, Ulf Ribacke

**Affiliations:** 1 Department of Microbiology, Tumor and Cell Biology, Karolinska Institutet, Solnavägen 9, SE-17165 Solna, Sweden; 2 Department of Learning, Informatics, Management and Ethics, Karolinska Institutet, Tomtebodavägen 18, SE-17177 Solna, Sweden; 3 Department of Cell and Molecular Biology, Karolinska Institutet, Solnavägen 9, SE-17165 Solna, Sweden; 4 Department of Cell and Molecular Biology, Uppsala University, Husargatan 3, SE-75237 Uppsala, Sweden

**Keywords:** Malaria, Parasite host response, Parasite biology, Small RNAs, RNA sequencing, Extracellular signalling molecules, Stress signalling

## Abstract

The lethal malaria parasite *Plasmodium falciparum* needs to constantly respond and adapt to changes within the human host in order to survive and transmit. One such change is composed of nutritional limitation, which is augmented with increased parasite loads and intimately linked to severe disease development. Extracellular vesicles released from infected red blood cells have been proposed as important mediators of disease pathogenesis and intercellular communication but whether important for the parasite response to nutritional availability is unknown. Therefore, we investigated the abundance and small RNA cargo of extracellular vesicles released upon short-term nutritional starvation of *P. falciparum *in vitro cultures. We show that primarily ring-stage parasite cultures respond to glucose and amino acid deprivation with an increased release of extracellular vesicles. Small RNA sequencing of these extracellular vesicles further revealed human miRNAs and parasitic tRNA fragments as the main constituent biotypes. Short-term starvations led to alterations in the transcriptomic profile, most notably in terms of the over-represented biotypes. These data suggest a potential role for extracellular vesicles released from *P. falciparum* infected red blood cells in the response to nutritional perturbations, their potential as prognostic biomarkers and point towards an evolutionary conserved role among protozoan parasites.

## Introduction

*Plasmodium falciparum* malaria represents a major global health burden with approximately 200 million cases and 600 thousand deaths annually^1^. Although elimination campaigns have significantly reduced infection, morbidity and mortality rates during the last decades, this positive trend has stagnated in recent years^1,2^. One of many reasons for these efforts to only be partly successful is the parasite’s ability to respond and adapt to treatments, which has led to drug resistance development towards antimalarials and difficulties to engineer vaccines with high efficacies^3,4^.

Besides withstanding drugs and vaccines, the parasite also needs to cope with other changes within the human host in order to survive and transmit. This is in particular true for the clinically relevant stage of the life cycle in which the asexual parasite resides and replicates within red blood cells (RBCs). During this stage, the parasite will experience natural variations in host status, metabolism and nutritional availability but also more grave nutritional perturbations as the disease progresses^5,6^. At the asexual stage, the parasite is fully dependent on glycolysis for its ATP production and scavenging of significant amounts of blood glucose from the host is therefore essential^7^. This also holds true for plenty of other nutrients^8^, including amino acids. Although the host RBC provides plenty of amino acids through the ubiquitous hemoglobin, the reservoir is insufficient as it lacks isoleucine^9^. Consequently, as the infection progresses, many disease manifestations are intimately linked to the metabolic requirements of the parasite. For example, hypoglycemia, metabolic acidosis and amino acid availability have been closely linked to the pathophysiological status and disease severity of malaria patients^10,11^. How the parasite responds and adapts to changes like these in order to survive is poorly understood. Therefore, increasing the knowledge of the mechanisms used by the parasite to cope with starvation and stress is important, as it would improve the understanding of the disease pathogenesis and possibly also provide new approaches for how to target and eliminate the parasite.

Extracellular vesicles (EVs) released from infected RBCs (iRBCs) have recently gained much attention and have been suggested to be important mediators of disease pathogenesis but also for intercellular communication and parasite adaptation^12,13^. The EVs and their content have been shown able to influence the host immune response by orchestrating cytokine release, trigger gametogenesis commitment and spread of drug resistance traits through horizontal gene transfer^14–16^. Additionally, previous analyses by RNA sequencing (RNA-seq) have revealed that EVs derived from iRBCs contain human miRNAs, which are shuttled to endothelial cells and thereby modulate regulation of gene expression and barrier properties of these^17,18^. Moreover, differences in the expression level of miRNAs in EVs have been linked to the host’s infection status and hemoglobin genotype^19,20^. Besides the relevance of the EV cargo for disease pathogenesis, recent studies have shown correlations between severe malaria, such as cerebral manifestations, and the amount of circulating EVs in patient plasma^21,22^. Thus, EVs released by iRBCs appear as important mediators of pathobiology, parasite adaptation and could possibly constitute prognostic or diagnostic biomarkers^15,23^.

Whether the release of EVs is involved in orchestrating the *P. falciparum* response to metabolic challenges is unknown. Interestingly, a recent study investigating how *Trypanosoma cruzi* responds to stress, revealed an increased release of EVs upon different stimuli, possibly indicating EV release to be a common mechanism for protozoan parasites to respond to environmental changes on a population level^24^. Here, we address the question whether also *P. falciparum* responds via EVs to clinically relevant stress in the form of nutritional starvation and show that the release of EVs from ring-stage iRBCs is augmented upon short-term starvation of glucose and amino acids. We also provide a comprehensive overview of the small RNA cargo of these EVs and reveal that the transcriptomic profile is altered upon growth perturbations.

## Results

### Short-term starvation induces an increased release of EVs from ring-stage cultures

**Figure 1:**
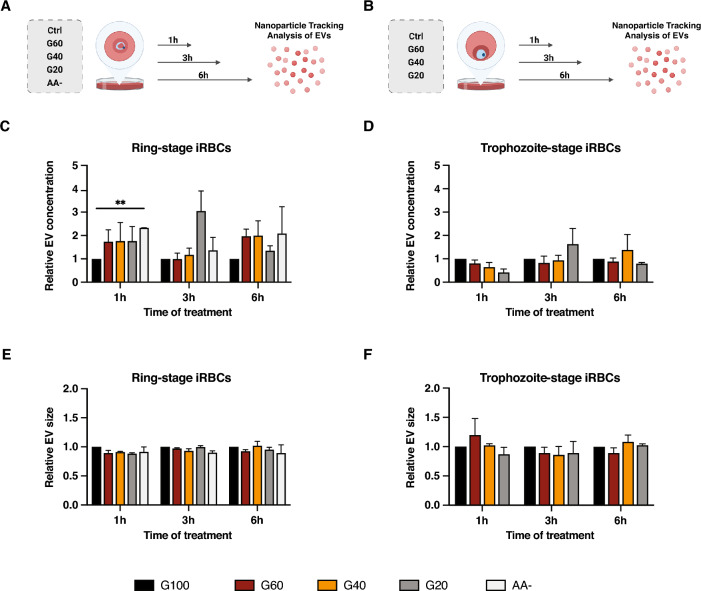
Short-term starvation induces an increased release of EVs from primarily ring-stage *P. falciparum* cultures. Schematic overview of the experimental procedure to generate nutritionally starved cultures containing (**A**) ring-stage or (**B**) trophozoite-stage *P. falciparum* cultures. Concentrations of EVs were determined by NTA after 1–6 h of exposure to different media compositions and were normalized to control for (**C**) ring-stage cultures starved of glucose and amino acids and (**D**) trophozoite-stage cultures starved of glucose. The size of the EVs from the same preparations were determined and similarly normalized to control for (**E**) ring-stage cultures and (**F**) trophozoite-stage cultures. For all plots (**C**–**F**), averages from three biological replicates are shown, except for the AA-treatment of ring-stage cultures, which was performed twice. Error bars represent standard error of the mean and asterisks indicate *p* values as determined by repeated measures (RM) two-way ANOVA with Geisser-Greenhouse correction in combination with Dunnett’s multiple comparison test (**p ≤ 0.01).

To investigate whether the release of EVs is altered upon nutritional starvation of iRBCs, we exposed tightly synchronized ring-stage (12 ± 2 h post invasion of RBCs (hpi)) (Fig. 1A) and trophozoite-stage (28 ± 2 hpi) (Fig. 1B) cultures to different glucose and amino acid concentrations for different durations of time. Purified EVs were analyzed by nanoparticle tracking analysis (NTA), which revealed rapid elevation of particle counts in ring-stage cultures upon amino acid deprivation (AA-) and when glucose was reduced to 20% of the normal concentration (0.4 g/l, G20) (Fig. 1C). Upon longer starvation (6 h), also milder glucose starvations (G40, G60) induced a higher abundance of released EVs. These findings were further corroborated by measuring total protein concentration and levels of selected transcripts previously shown to be part of the EV cargo from *P. falciparum* cultures^17^. Consistent with the increased particle concentrations, protein levels and selected transcripts of parasite origin were shown to be elevated upon starvation (Fig. S1A,B). Intriguingly, the trend of elevated EV release upon stress of ring-stages was not as apparent in glucose starved trophozoite-stage cultures (Fig. 1D) and in neither case did perturbations of culture conditions lead to any significant differences in average size of EVs (Fig. 1E,F).

### The cargo of EVs consists of small RNA and most abundantly human miRNA

**Figure 2:**
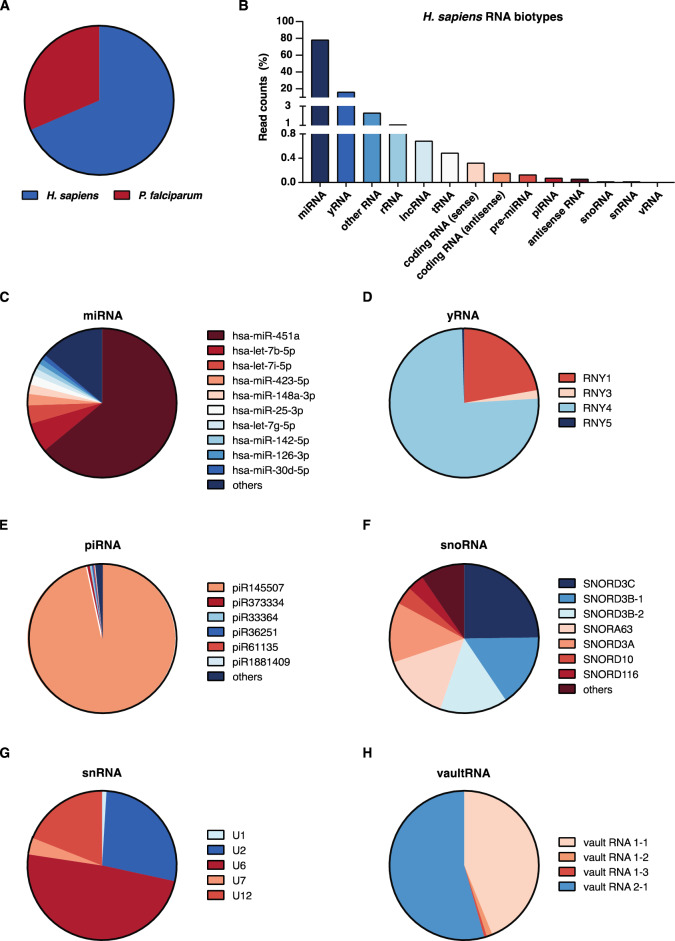
The human RNA cargo of EVs from *P. falciparum* parasite cultures is dominated by miRNA. (**A**) Overall read distribution by species from small RNA-seq of EVs from *P. falciparum* cultures. (**B**) Distribution of biotype representation as % of all reads from all sequenced EV preparations. (**C**–**H**) Pie charts depicting distributions of the most abundant human reads among individual members in biotypes for (**C**) miRNAs, (**D**) yRNAs, (**E**) piRNAs, (**F**) snoRNAs, (**G**) snRNAs and (**H**) vaultRNAs.

The notion that the release of EVs is induced from iRBCs upon starvation probed us to explore whether also the RNA cargo was altered. Therefore, RNA was collected from large ring-stage cultures in triplicates, either grown in normal medium or in medium devoid of amino acids or with lowered glucose levels for 4 h. Consistent with previous findings^17^, EVs were shown to contain mainly small RNAs and that iRBCs release more EVs than uninfected RBCs (uRBCs) (Fig. S2). In line with these findings, small RNA sequencing (RNA-seq) libraries were prepared and sequenced. Different alignment approaches were explored to enable mapping and discrimination of parasite and human reads. In our hands, using default parameters, we found that the use of TopHat provided superior separation of the reads compared to STAR, possibly due to the short read lengths (Fig. S3). Using the most discriminatory mapping approach, the overall distribution of reads was shown to be of roughly two thirds human and one third parasite origin (Fig. 2A).

The human reads were further assigned to different RNA biotypes, which revealed a wide spectrum of classes but with miRNAs to be the most abundant (almost 80%), followed by yRNA (16.3%) (Fig. 2B). All other identified biotypes contributed substantially less to the overall cargo, which besides RNA derived from coding sequences in sense and antisense direction, included many other non-coding RNA populations, such as lncRNA (0.68%), tRNA fragments (0.49%), pre-miRNA (0.13%), piRNA (0.07%), antisense RNA (0.06%), snoRNA (0.015%), snRNA (0.013%) and vaultRNA (0.003%) (Fig. 2B).

The over-representation of miRNAs in EVs from ring-stage iRBCs is consistent with previous findings from late-stage cultures^17^. This class of transcripts has been suggested as important in the interaction between the parasite and the host and differential expression has been implicated in severe disease development^25^. For example, miRNAs have been shown to alter barrier properties of human endothelial cells to the parasite’s advantage^18,26^ but also to influence the transcriptional and translational machinery of the parasite, displaying a multi-functional role in malaria pathogenesis^27,28^. The pathophysiological relevant miR451a was the most abundant transcript of all in our dataset (Fig. 2C), a miRNA that has been associated with alteration of parasite growth and sickle cell disease^19,27^. Other miRNAs identified at high abundance and of potential pathophysiological importance were hsa-let-7b-5p, hsa-let-7i-5p and hsa-miR423-5p (Fig. 2C). On the contrary, pre-miRNAs were of low abundance and only represented by 0.13% of all human originated reads and with a more equal distribution than their mature counterpart (Fig. S4A). The most expressed pre-miRNA was let-7b (27.88%) followed by mir-451a (12.07%), mir-451b (9.42%) and mir-182 (7.02%).

The second most abundant RNA biotype derived from human origin was yRNAs, a small noncoding RNA class which is structurally associated with the Ro60 complex and functionally thought to be involved in DNA replication, RNA stability and cellular stress responses^29–31^. There are four yRNAs known in humans: RNY1, RNY3, RNY4 and RNY5. In our samples, we could identify fragments of all four with RNY4 being the most abundant (75.5%) **(**Fig. 2D). Variable distribution of individual family members was observed for the other, less represented biotypes. Although over 20 piRNAs, the class of small silencing RNAs serves as guide RNAs for a subset of argonaute proteins called PIWI proteins, were detected, piR145507 stood for almost 97% of all reads (Fig. 2E). For the small nucleolar RNAs (snoRNAs), another class of small RNAs with gene regulatory implications^32,33^, different variants of SNORD3 were most abundant together with SNORD10, SNORD116 and SNORA63 (Fig. 2F). Distinct distributions were also observed among the spliceosomal RNAs (Fig. 2G) and vaultRNA fragments (Fig. 2H). Lastly, only a small fraction of human derived tRNAs could be detected, with the majority of reads (96.2%) assigned to Gly-GCC tRNA (Fig. S4B).

### EV resident parasite transcripts are dominated by tRNA fragments

**Figure 3:**
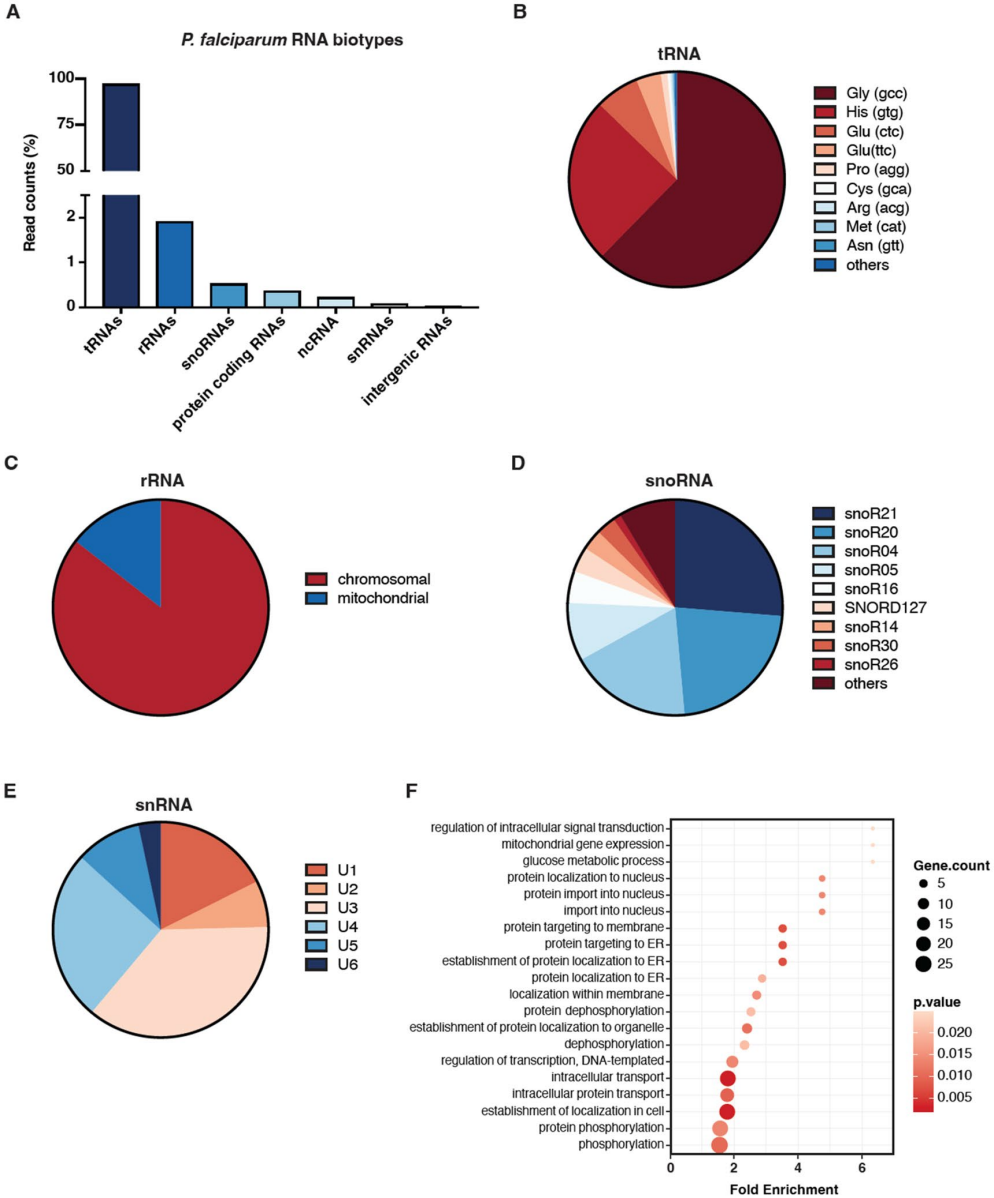
The parasitic RNA cargo of EVs from *P. falciparum* cultures is highly enriched in tRNA fragments. (**A**) Distribution of parasite derived reads in biotypes as % of all reads from all sequenced EV preparations. (**B-E**) Pie charts depicting distributions of the most abundant parasite reads among individual members in biotypes for (**B**) tRNAs, (**C**) chromosomal and mitochondrial rRNAs, (**D**) snoRNAs and (**E**) snRNAs. (**F**) GO term enrichment analysis of biological processes for all reads originating from protein coding genes in *P. falciparum*. The top 20 significant categories (all with *p* ≤ 0.05) are displayed.

In stark contrast to the representation of human derived transcripts, the overarching majority of parasite reads was assigned to tRNA fragments (96.89%). We could further identify rRNAs (1.91%), snoRNAs (0.52%), protein coding RNAs (0.36%), other ncRNAs (0.22%), snRNAs (0.07%) and intergenic RNAs (0.03%) (Fig. 3A). The tRNA-derived fragments have recently gained increased attention in *P. falciparum* and other protozoan parasites and have been proposed to be involved in mediation of cellular stress responses and posttranscriptional regulation^34–36^. They have also been identified as a dominant RNA cargo of EVs released by *Trichomonas vaginalis* grown in serum free medium^37^. Here, we identified Gly^GCC^ as the most abundant tRNA with 62.32% of the reads. Other highly abundant tRNAs were His^GTG^ and Glu^CTC/TTC^. Interestingly, the first four tRNAs accounted for more than 95% of all tRNA reads identified from parasitic origin (Fig. 3B).

Among the remaining biotypes, ribosomal RNAs were the most abundant. Unlike previously reported^17^, the ribosomal RNAs identified in our dataset were mostly derived from chromosomal origin (Fig. 3C) of which the overarching majority originated from 28s rRNA (Fig. S5). For snoRNAs, that are mainly associated with ribosomal biogenesis but also other gene regulatory processes^38^, we could identify over 30 different types. Of these, snoR21 (26.29%) and snoR20 (22.29%) were the most abundant closely followed by snoR04, snoR05 and snoR16 (Fig. 3D). Small nuclear RNAs are essential mediators of splicing processes in eukaryotes. The highly conserved spliceosome in eukaryotes consists of five snRNAs: U1, U2, U4, U5, and U6^39^. We could identify all five spliceosomal RNAs in our *P. falciparum* dataset with U4 being the most abundant (25.6%) in addition to the highly expressed, non-spliceosomal U3 RNA (Fig. 3E).

Besides the noncoding RNAs, we could identify 776 protein coding transcripts from *P. falciparum*. Most of those genes related to transport processes and protein phosphorylation in the parasite when analyzed by GO-term analysis (Fig. 3F). The most prominent transcripts were from genes encoding early transcribed membrane proteins, the ring-infected erythrocyte surface antigen, subunits of the PTEX complex, but also heat shock proteins and members of the ALBA family. Thus, the transcriptomic landscape of the EVs revealed a broad range of structural RNAs, but in particular small RNAs functionally associated to gene regulatory activities were of high abundance, such as human miRNAs and parasitic tRNA fragments.

### Short-term starvation alters the EV RNA cargo

**Figure 4:**
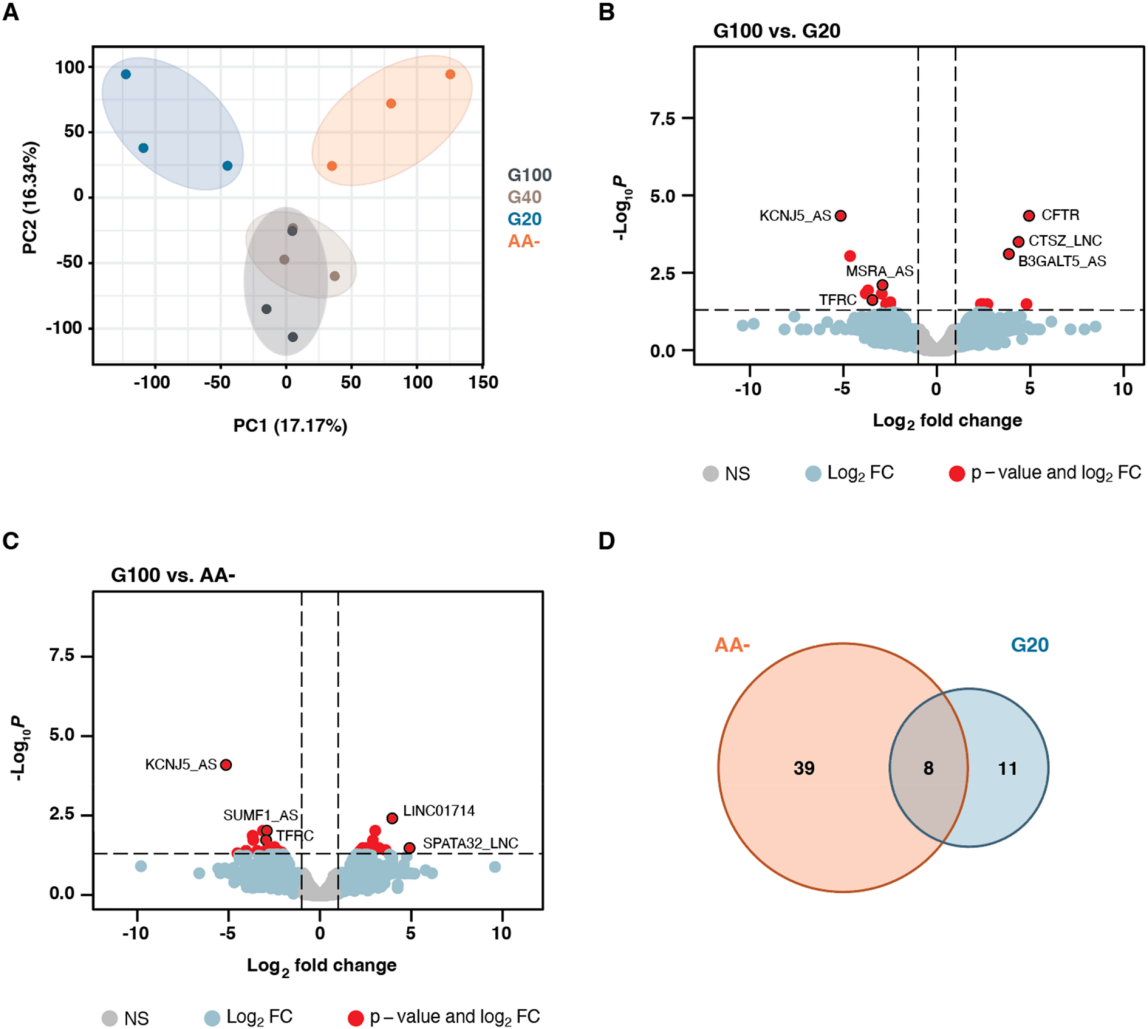
The small RNA cargo of EVs changes upon nutritional starvation of *P. falciparum* cultures. (**A**) Principal component analysis shows similar variances between the biological triplicates of each treatment condition and identifies a global separation of G20 (blue) and AA- (orange) from control (dark grey) and G40 (beige) treatments. (**B-C**) Volcano plots of differential expression compared to control for (**B**) G20 treated cultures and (**C**) AA- treated cultures. A relatively small number of differentially expressed genes (FDR adjusted *p* ≤ 0.05 and Log_2_FC ≥ 1 or ≤  − 1, red) were identified among the other genes displaying Log_2_FC ≥ 1 or ≤ − 1 (light blue) or smaller changes in expression (light grey). The most significantly expressed genes are highlighted (outlined in black) and annotated. (**D**) Venn diagram depicting the overlap of the significant differentially expressed transcripts (FDR adjusted *p* ≤ 0.05 and Log_2_FC ≥ 1 or ≤ − 1) between AA- (orange) and G20 (light blue) treatments.

After investigating the overall RNA cargo of EVs, we explored potential differences upon nutritional starvation. Biological replicates of the different starvations clustered together in a principal component analysis by PC1 (17.17%) and PC2 (16.34%) (Fig. 4A). While the G40 treatments clustered closely to the control group (G100), the two harsher starvations G20 and AA- gave distinct clusters. Therefore, these were further investigated in downstream analyses. Differential gene expression (DGE) analysis of G20 and AA- in comparison to the control group (Fig. 4B,C, Data file S1) revealed 19 and 47 differentially expressed transcripts respectively (Fig. 4D). The majority of differentially expressed transcripts were of human origin and were lncRNAs or of unknown genomic origin (labeled as other RNA, Data file S1). Both treatment groups shared eight differently expressed transcripts (see Data file S1) including the downregulated antisense lncRNA towards the methionine sulfoxide reductase A (MSRA) gene, which is implicated in cellular stress and associated to mediate the repair of oxidative damage to proteins^40^. Another differently expressed transcript of interest was the transferrin receptor derived TFRC-203, which belongs to the group of transferrin receptors that play an important role in the regulation of iron intake and erythropoiesis^41^. Both G20 and AA- displayed slightly more downregulated than upregulated transcripts (Fig. S6A) whereas the representation of sense vs. antisense transcripts was the same (Fig. S6B). The two parasitic tRNAs Pro^CGG^ and Gln^TTG^ were significantly downregulated in amino acid deprived conditions according to our DGE analysis.

### Amino acid starvation results in altered miRNA profiles and reduced tRNA fragments in EVs

**Figure 5:**
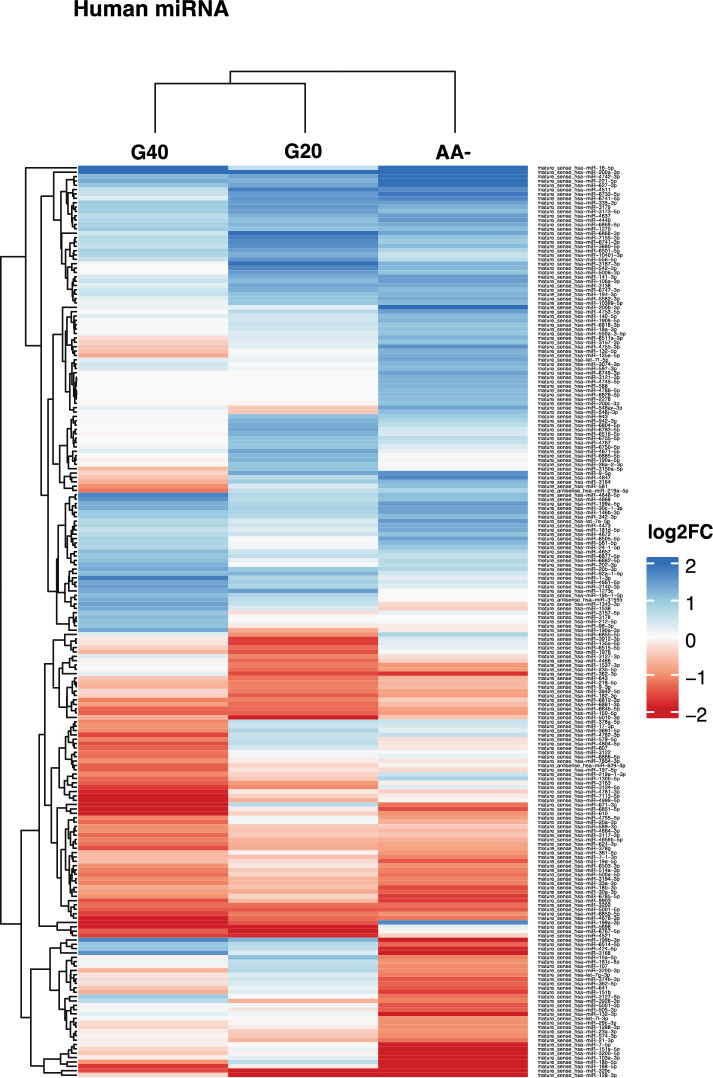
The human miRNA repertoire in EVs from *P. falciparum* cultures changes with degree and type of nutritional starvation. Correlation heatmap of normalized read counts for all mature miRNAs with Log_2_FC ≤ − 1 or ≥ 1 for the different starvation conditions compared to control. Each row represents a unique mature miRNA with coloring based on average Log_2_ FC from three biological replicates and columns are represented by the different treatment conditions.

To further broaden our understanding of potential cargo changes, we also employed a less conservative approach and investigated human and parasitic transcripts with a Log2 FC ≤ − 1 or ≥ 1 for all treatment groups. Thereafter we compared the miRNAs in a clustered heatmap (Fig. 5). While glucose starvation led to less changes and clustered together, the AA- treatment group displayed a more distinct miRNA profile. Upon amino acid starvation, more miRNAs were either up or down regulated and among them miR140-5p, miR15a-5p and miR-let7i, which have previously been assigned possible biomarker roles in the context of malaria infection^42,43^.

**Figure 6:**
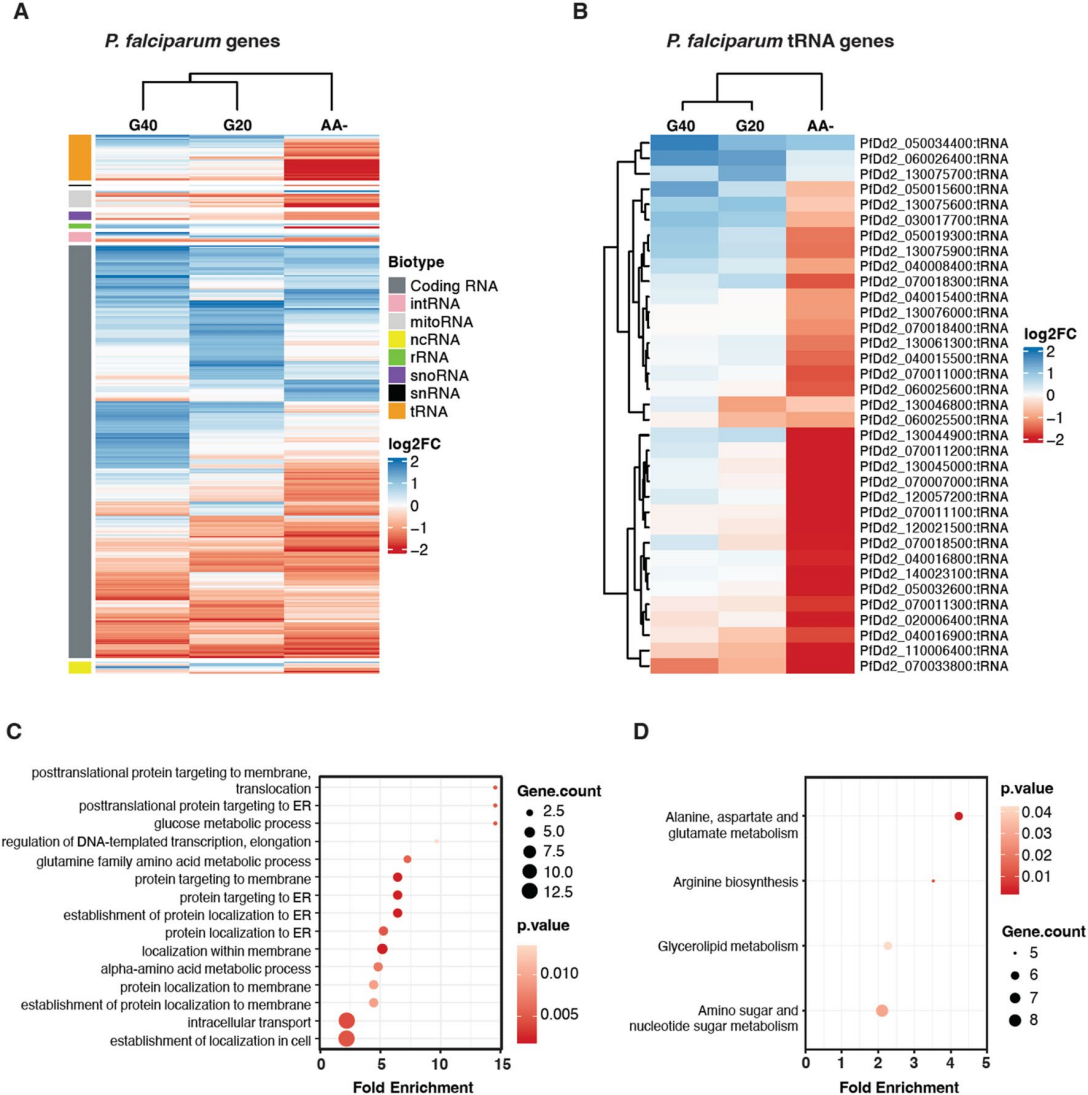
The parasitic small RNA cargo of EVs is correlated to the degree and type of nutritional starvation. (**A**) Supervised correlation heatmap of normalized read counts for all parasitic transcripts displaying Log_2_ FC ≤ − 1 or ≥ 1 from three biological replicates of the different starvation conditions compared to control. Rows were ordered based on transcript biotypes whereas columns were clustered based on treatment conditions. (**B**) Correlation heatmap of all parasitic tRNAs fragments displaying Log_2_ FC ≤ − 1 or ≥ 1 from three biological replicates of starvations. Rows represents unique tRNA fragments and columns the different starvation conditions. (**C**) GO term enrichment analysis of biological processes for all parasite transcripts with an average Log_2_ FC ≤ − 1 or ≥ 1. The top 15 GO terms (all with *p* ≤ 0.05) are displayed. (**D**) KEGG pathway analysis for all parasite transcripts with an average Log_2_ FC ≤ − 1 or ≥ 1. All significantly enriched (*p* ≤ 0.05) pathways are displayed.

Next, all parasitic transcripts with a Log2 FC ≤ − 1 or ≥ 1 were analyzed in a similar manner. We could observe a similar picture as for the human cargo, namely a more distinct separation of amino acid deprived from glucose starved samples (Fig. 6A). All previously assigned biotypes were represented in a clustered heatmap with tRNA fragments displaying the largest and most consistent differences. The absolute majority of tRNA fragments in the AA- group were downregulated, with Gln^TTG^, Met^CAT^ and Lys^CTT^ displaying the largest difference in expression (Fig. 6B). As many transcripts from protein coding genes displayed a Log2 FC ≤ − 1 or ≥ 1 we subjected those to a GO term enrichment analysis for biological processes (Fig. 6C). While most were involved in “localization within membrane” and “protein targeting”, we could also identify processes associated to metabolism such as “glucose metabolic process” and “alpha-amino acid metabolic process”. To further explore these findings, we conducted a KEGG pathway analysis for the same set of transcripts and identified “amino sugar and nucleotide sugar metabolism” as the most enriched pathway with the highest gene count (n = 8) (Fig. 6D).

Taken together, the abundance and the cargo of EVs released by iRBCs appear responsive to short-term starvation, with transcripts of both human and parasite origin being perturbed. Future scrutiny of the role of the RNA cargo could provide a better understanding of the role of these EVs in the parasite’s adaptation to environmental changes and in disease pathogenesis.

## Discussion

Here we provide the first evidence of *P. falciparum* iRBCs responding to nutritional starvation by a rapid and augmented release of EVs and thereby highlight their potential role in disease pathogenesis, parasite adaptation and potential as prognostic biomarkers. We also show that this release appears to be stage dependent, with enhanced release from ring-stage iRBCs, at least in response to glucose starvation and that the cargo changes specifically with different types of starvation. This suggests a potential nutrient sensing role for EVs in the mediation of cellular response on a population level.

The relationship between altered EV levels in patient plasma upon *Plasmodium* infection has been reported previously and increased EV numbers have been associated to disease severity^23,44,45^. This could be explained by higher infection loads, as higher parasitemia is also generally correlated to worsened pathobiology of the patient^46,47^. However, with an increased parasite load, the metabolic and nutritional constraints on the parasites increase as the consumption rates of nutrients, such as glucose and amino acids, often reaches levels to render the patients nutritionally deprived^10,11^. Thus, it is plausible that the observations done in patients are a result of both, namely a higher biomass of iRBCs able to release EVs in conjunction with the release being augmented in response to cellular stress. In favor of different cellular stresses being important stimuli for the release of EVs are also previous findings in other protozoan parasites. Acidic pH and low temperature induce EV release in *T. cruzi*^24^ and early studies on *Leishmania* have revealed EV release to be increased as a response to elevated temperatures resembling the change in environments from the vector to the human host^48^. Taken together, our data and the results of others suggest a potentially conserved role of EV release from protozoan parasites upon different types of stress, potentially to ensure parasite survival and/or transmission. Still, many underlying modalities of an increased EV release upon stress remain to be elucidated.

The majority of initial in vitro studies on *P. falciparum* derived EVs were conducted on late-stage parasite populations^12,17,18,49,50^, whereas recent studies have focused more on ring-stage parasites and elucidating stage dependent differences^20,51–53^. Together with analyses of EVs from malaria patient samples, they have led to an improved understanding of the cargo and their role in parasite biology and disease pathogenesis^12–15,54–56^. Here we explored both ring- and trophozoite-stage cultures and detected increased EV numbers mainly from ring-stage iRBCs. While ring-stage parasites for long were considered a rather inactive stage, this stage has gained more attention as an important adaptive part of the parasite’s life cycle, perhaps best illustrated by the role in artemisinin drug resistance^57–59^. Only little is known about the functional implications of ring-stage derived EVs but a recent study demonstrated that those EVs can carry PfEMP1, a crucial protein in parasite virulence, and have the potential to alter the transcriptomic landscape of monocytes^51^. Taken together, this suggests that the ring stage of *P. falciparum* has a potentially larger role in the mediation of stress responses and disease pathogenesis, possibly through the release of EVs, than originally thought.

The small RNA landscape of ring-stage iRBC derived EVs correlated in general well with what was previously reported from a similar small RNA-seq study conducted on late-stage cultures^17^. There were however some differences in representations of both biotypes and specific transcripts, in particular in relation to *P. falciparum* transcripts. These differences could be due to variations in parasite stages used and/or the processing of the RNA-seq data, as databases of human and *P. falciparum* transcripts change over time and so does the possibility to map transcripts accurately and discriminatorily. Highly consistent between the studies were however the large fraction of the cargo constituted by human miRNAs. Interestingly, transcriptomic changes in the miRNA profile of EVs have previously been observed in patient samples, which potentially allows for a distinction of *P. vivax* and *P. falciparum* infections based on their miRNA profile^60^. In addition, differences in the miRNA profile have been linked to disease severity and genotypic background of the patient with regard to the protecting sickle cell genotype^19,25,27,61^. Our data indicated alterations in miRNA expression most prominently observed upon amino acid starvation whereas not as pronounced during glucose starvation. Importantly, these experiments were conducted with the same batches of RBCs and the results should therefore not be influenced by human genotypic differences. Still, whether the observed changes in miRNA profile would affect disease progression or parasite behavior in vivo is yet to be elucidated. However, EV derived human miRNAs have previously been shown to act regulatory in endothelial cells due to the accompanied delivery of the Argonaute 2 protein^18^. It is therefore possible that the starvation induced changes in miRNA expression could lead to differences in gene regulation in both host cells and parasites^17,18,28^.

When scrutinizing the parasite derived RNA cargo, tRNA fragments accounted for the absolute majority of reads, independently if parasite cultures were starved or not. The over-representation of tRNA fragments has also been observed in EVs released from *T. vaginalis*, where almost 90% of all reads were derived from tRNAs ^37^. Studies on the transcriptomic cargo of EVs released from other protozoan parasites such as *Leishmania spp.*^62^, *Trypanosoma cruzi* ^63^and *Giardia intestinalis*^64^ have similarly demonstrated that tRNA fragments are a dominant biotype. This could suggest that the packing of these potentially gene regulatory tRNAs in EVs is a conserved mechanism among protozoans. In addition, our study revealed Gly^GCC^, His^GTG^, Glu^CTC/TTC^ and Pro^AGG^ to be the most dominant tRNA fragments, which is similar to the observations in other protozoans where Gly and Glu tRNA fragments were among the most abundant^62–64^. Transcriptomic studies of EVs from different protozoans also suggest that the RNA cargo of EVs is independent of the transcriptomic profiles of the cells from which the EVs originate^17,62,63^. Comparing the tRNA profile of our dataset to the cellular tRNA profile of *P. falciparum* iRBCs^65^, it is tempting to speculate that this might be the case for *P. falciparum* as well. Still, none of the efforts to elucidate protozoan RNA cargo of EVs to date have employed specialized tRNA sequencing methodologies, mainly as these require RNA quantities that are difficult to obtain from EV preparations. As tRNAs are heavily modified and therefore difficult to sequence with regular approaches, it is plausible that the tRNA profiles of EVs are even more complex than shown here. Even so, the striking abundance of tRNAs in the absence of specialized sequencing, the discordance between cellular and EV transcriptomes and the intriguing similarities between different protozoans, point toward an evolutionary conserved but yet elusive role of tRNA fragments in parasite derived EVs.

The most striking differences observed in EV cargo from the different starvations were the ones of parasite tRNA fragments and human lncRNAs. The latter have been proposed to be involved in gene regulation of recipient cells and immunomodulation based on studies of EVs in other cellular systems^66^. Many parasitic tRNA fragments were downregulated upon amino acid starvation with the two significantly expressed being Pro^CGG^ and Gln^TTG^. In contrast, nutrient starvation also led to an increase of certain tRNA fragments, specifically of Arg^TCG^, Ser^TGA^ and Trp^CCA^ independent of type of condition. Interestingly, glucose starvation exclusively led to an increase in Leu^TAG^ and Ile^AAT^ tRNA fragments, which could not be observed upon amino acid starvation. What this means for the biology of the parasite or pathogenesis of the disease remains to be elucidated, but it is tempting to speculate that parasitic tRNAs might be involved in parasite stress sensing^65^. Together with the remaining high abundance of small RNAs with gene regulatory potential, it is likely that the EV cargo overall plays a significant role in the host-parasite or parasite-parasite interaction.

Taken together, the existing body of EV studies in protozoan infections and our newly presented data possibly suggest that EVs are used as a common tool for intercellular communication in protozoan parasite infections^16,54,67–69^. Even though the role of the parasitic EV stress response remains unknown, our study provides the first evidence of EV abundance and small RNA cargo of *P. falciparum* derived EVs changing upon nutritional starvation.

## Methods

### Parasites, in vitro culture and media compositions

The *P. falciparum* strain Dd2 was cultured according to standard procedures^70^ with minor modifications. In brief, parasites were cultured in human O + RBCs at a hematocrit of 4% in culture medium consisting of in-house prepared RPMI 1640 (see below) supplemented with 0.025 mg/ml Gentamycin (Gibco Life Technologies) and 0.5% Albumax (Gibco Life Technologies). Cultures were maintained in suspension^71^ on an orbital shaker (50 rev/min.) at 37 °C and in a constant microaerophilic environment of 1% O_2_, 5% CO_2_ and 94% N_2_. Different RPMI1640 compositions were prepared to be used in media for regular parasite culture and starvation experiments. For continuous culture and for glucose starvation, RPMI 1640 powder with l-glutamine and without glucose (USBiological #R9010) was reconstituted to 1 × in ddH2O and supplemented with 367 μM hypoxanthine, 20 mM HEPES, 28 mM NaHCO_3_ (all Sigma-Aldrich). Glucose (Sigma-Aldrich) was thereafter added at four different concentrations; 2 g/l for continuous culture and controls (G100), 1.2 g/l for 60% (G60), 0.8 g/l for 40% (G40) and 0.4 g/l for 20% (G20) of normal glucose. For medium depleted of amino acids, RPMI1640 powder without glucose and amino acids (USBiological #R9010-01) was reconstituted the same way but with glucose added to only one concentration (2 g/l). Parasite cultures were regularly synchronized by treatments with 5% sorbitol as described previously^72^.

### Small-scale starvation experiments and EV purification

Cultures with tightly synchronized ring- (12 ± 2 h post invasion (hpi)) or trophozoite-stage (28 ± 2 hpi) iRBCs were subjected to media with different glucose levels or devoid of amino acids at different scales and duration before iRBCs and supernatants were separated for further processing. Initially, ring-stage iRBCs were exposed to G100, G60, G40, G20 and AA- media for a total of 6h and harvested at regular time intervals. Supernatants were separated from cells through a series of 15 min long centrifugations with incremental force (600, 1600, 3200 and 10,000×*g*) at 4 °C, with supernatant transferred into a new sterile tube in between each centrifugation. After realizing what durations of starvation and media compositions that altered the release of EVs from ring-stage iRBCs, trophozoite-stage iRBCs were exposed to G100, G60, G40 and G20 for 6 h. Supernatants were collected at regular time intervals and purified in the same manner as the ring-stage experiments. All the final samples were stored at − 20 °C until further analysis by NTA, RT-qPCR, Bradford protein quantification and Western blot (see details below).

### Nanoparticle tracking analysis

EVs from purified culture supernatants were analyzed for their size and particle concentration using a NanoSight NS500 instrument and NTA 2.3 analytical software. Prior to analysis, samples were diluted in 0.22 μm filtered PBS to an appropriate concentration (10–100 particles/frame). Each sample was recorded for at least five 30 s videos in light scatter mode with a camera level of 11–13. Sample analysis settings within the software were kept the same for all measurements (screen gain 10, detection threshold 7).

### Protein quantification (Bradford)

The concentration of proteins in EVs was determined using a Bradford kit (Sigma-Aldrich) with bovine serum albumin (BSA, Sigma-Aldrich) as a standard. Lysates to be analyzed were prepared using equal amounts of Laemmli buffer lacking bromophenol blue and were further diluted during the assay (1:31) to prevent buffer constituents to be incompatible with the quantification. The assay was carried out according to the instructions of the manufacturer and samples were measured at a wavelength of 545 nm after an incubation time of 40 min at room temperature.

### SDS-PAGE and Western blot analysis of EV purity

To ensure that the EVs were of adequate purity and devoid of cellular and/or soluble, extravesicular material, preparations were analyzed by SDS-PAGE and western blot (Fig. S7). Individual preparations from (A) purified EVs, (B) the supernatant remaining after purification of EVs and (C) cells were diluted in Laemmli sample buffer (Bio-Rad) and boiled for 5 min at 97 °C. Proteins were separated by size on Any kD Mini-PROTEAN TGX Stain-Free Precast SDS-page gels (Bio-Rad). Proteins were transferred onto a nitrocellulose membrane using a TransBlot Turbo Transfer system (Bio-Rad). Membranes were blocked in PBS with 0.05% Tween20 (PBS-T) supplemented with 3% BSA and 3% milk powder at room temperature for 1h. Primary antibodies against α-Stomatin (1:2500, sc-376869, Santa Cruz) and α-Band3 (1:12,500, sc-133190, Santa Cruz) were used to detect proteins known to be resident in EVs from human RBCs, whereas α-HRP2 (1:500, AB_2539620, Thermo Fisher) and α-HSP70 (1:10,000, SPC-186D, StressMarq Biosciences Inc.) were used to detect exported and cell-internal parasite proteins respectively. Membranes were incubated with primary antibodies diluted in PBS-T with 3% BSA for 2h at room temperature before being washed 4 × 5min in PBS-T prior to the addition of the secondary antibodies (GE Healthcare), which were HRP-conjugated goat anti-rabbit (1:25,000) and rabbit anti-mouse IgG (1:10,000) diluted in PBS-T with 3% BSA. After 1h of incubation at room temperature, blots were washed 5 × 5min with PBS-T and immunoreactive bands were detected using Pierce™ ECL (Thermo-Fisher) according to the instructions of the manufacturer before visualization with a ChemiDoc XRS + system (Bio-Rad).

### Large-scale starvation experiments and EV purification for RNA seq analysis

Large parasite cultures (50 ml) were exposed to G100, G60, G40, G20 and AA- media for 4 h. The supernatant was harvested and subjected to a series of centrifugations at 4 °C; 600×*g* for 20 min, 1600×*g* for 15 min and 3200×*g* for 15 min with supernatant transferred into a new sterile Falcon tube in between each spin. The resulting supernatant was filtered through a sterile 0.22 μm PES filter unit to remove any larger particles. Next, the supernatant was transferred to 100 kD Millipore tubes (Merck) and centrifuged for 20 min at 4000×*g* at 4 °C. The retentates containing the purified EV samples (200 μl) were thereafter diluted and lysed in TRIzol reagent (1:5, Thermo Fisher), snap-frozen and stored at − 80 °C until further processing.

### RNA isolation

For RNA isolation, the EV TRIzol lysates were thoroughly mixed with 160 μl of chloroform and centrifuged at 16,000×*g* for 10 min at 4 °C. The aqueous phase was precipitated overnight at − 20 °C with 0.1 × V of 3 M sodium acetate pH 5.5, 1 × V of prechilled isopropanol and 0.025 × V of SuperaseIn RNase inhibitor (Invitrogen™). RNA was pelleted at 16,000×*g* for 30 min at 4 °C, washed with 500 μl of prechilled 75% ethanol, dried at room temperature and dissolved in 85 μl DEPC treated nuclease free water (Thermo Fisher). Next, RNA was treated with DNase for 20 min at room temperature by adding 2.5 μl TURBO™ DNase, 10 μl of 10X TURBO™ DNase buffer (both from Invitrogen™) and 2.5 μl of SuperaseIn (Invitrogen™) in order to remove DNA contaminations. After DNase treatment, RNA was purified using the NucleoSpin miRNA Kit (Macherey–Nagel) without size fractionation, according to the instructions of the manufacturer. The high retention of RNA on the columns of the kit allows for sequential purification without any apparent loss of RNA (not shown). RNA quantity and quality were assessed with the Qubit™ HS RNA assay kit and using the RNA 6000 Pico kit (Agilent) and a Bioanalyzer 2100 (Agilent). RNA was snap frozen on dry ice and stored at − 80°C until further use.

For interrogation of select transcripts by qPCR (see below), small scale RNA extractions were performed using the Direct-zol RNA MicroPrep (Zymo) according to manufacturer’s instructions.

### Reverse transcription and qPCR

Identical volumes of RNA from G100, G20, and AA- (10 μl each) were used for reverse transcription. RNA was denatured for 5 min at 65 °C in the presence of 2.5 μM oligo d(T)12–18 (Invitrogen™), 2.5 μM random decamer primers (Invitrogen™) and 1 U/μl SUPERase RNase Inhibitor (ThermoFisher). Reaction was snap-cooled on ice and 1 × SSIV Buffer, 5 mM DTT, 20 U/μl SUPERase RNase Inhibitor, 0.5 mM dNTP mix and SuperScriptTM IV Reverse Transcriptase were added. Samples were incubated at 25 °C for 5 min, 55 °C for 30 min and 75 °C for 15 min and stored at 4 °C until usage. Reactions with omitted SuperScriptTM IV Reverse Transcriptase were performed in parallel to be able to assess whether contaminating gDNA was present in down-stream qPCR amplifications. All incubations were performed in a ProFlex™ PCR System (Applied Biosystems™).

RT-qPCR analysis was performed in technical triplicates for each sample and primer pair. Genes of interest were investigated using the following primer pair against Pf_18s (F: 5′-GCTCTTTCTTGATTTCTTGGATG-3′ and R: 5′-AGCAGGTTAA-GATCTCGTTCG-3′) and Pf_28s (F: 5′-ACTGAAACGGACAAGGGGAAT-3′ and R: 5′- GCAGAAATCACATTGTGTTAATACCA-3′). A final volume of 10 μl was used per well containing 1μl of cDNA, 5 μl SsoAdvanced Universal SYBR Green Supermix (Bio-Rad) and 300 nM of primer. Amplifications were performed in a CFX384™ Real-Time PCR Detection System (Bio-Rad) with an initial denaturation of 10 min at 95 °C and 45 cycles of 95 °C for 15 s and 60 °C for 60 s. Gene expression levels in starved parasite cultures were computed as fold-change relative to levels in untreated controls. Final dCT values represent the mean of three biological replicates.

### RNA library preparation and RNA sequencing

RNA-seq libraries were prepared by using the NEBNext® Multiplex Small RNA Library Prep Set for Illumina® (NEB #E7580) according to the instructions of the manufacturer. Libraries were quantified using the Collibri™ Library Quantification kit and quality of libraries was determined by Agilent High Sensitivity DNA kit on the 2100 bioanalyzer system. RNA sequencing (1 × 75 cycles) was performed on the Nextseq 550 high output mode (Illumina).

### RNA-seq data processing

Raw sequence reads were first subjected to a quality control using FastQC (v0.11.8). Afterwards, adapter sequences were trimmed, and low-quality bases were removed using Trimmomatic SE (v0.39). Reads below 15nt in length were filtered out, followed by a second quality control using FastQC. In order to identify reads of human origin, reads were aligned against the Dd2 genome (PlasmoDB-54_PfalciparumDd2) using TopHat (v2.1.1) and Bowtie 2 (v 2.2.5) to remove *Plasmodium* reads. The remaining reads were annotated using the sRNA bench pipeline^73^. Briefly, the pipeline implemented a hierarchical sequence mapping strategy on the human genome (GRC v.38) with different reference databases, among them miRBase (release 22.1), using Bowtie2 (v 2.2.5) with parameters that allowed for one mismatch and seed length for alignment of 17. The alignments were processed using Samtools (1.9) and seqtk (1.3-r106). A count file with the results of the single alignment output files of sRNAbench was created. *Plasmodium* reads were identified by mapping trimmed reads first against the human reference genome (GRC v.h38) using TopHat and Bowtie 2 to filter out human reads. Reads that did not align against the human genome were kept and mapped against the Dd2 genome using TopHat and Bowtie 2. *Plasmodium* reads that could not be annotated using the Dd2 genome (“no feature reads”) were subjected to another round of alignment using TopHat and Bowtie 2 against the 3D7 reference genome (PlasmoDB-59_Pfalciparum3D7) to allow for annotation of reads mapping to UTR regions that were only annotated in the 3D7 genome. The remaining unannotated reads were manually annotated using the BLAST function in PlasmoDB^74^ and received a de novo annotation in case of intergenic hits (labelled as INT_xx). 3D7 annotated reads were converted back to Dd2 Gene IDs unless a Dd2 Gene ID was unavailable. In those cases, reads were de novo annotated (nc_xx for non-coding RNA, MITO_xx for mitochondrial genes). Mapped reads were quantified using htseq-count in union mode. Both human and *Plasmodium* count data was filtered for low read counts. Only genes with read counts ≥ 2 in at least 2 out of 3 replicates prior to normalization were used. The different datasets were combined using in-house python scripts.

### RNA-seq data normalization and differential expression analysis

To account for the mean–variance relationship inherent in RNA-seq count data and improve the comparability of gene expression levels across samples, a variance stabilizing transformation (VST) was applied using the DESeq2 package (version 1.36.0) in R. Given the possibility of technical artifacts or batch effects confounding the biological signal in the data, we employed the removeBatchEffect function from the limma package (version 3.52.4) to correct for such effects^75^. To assess the association between gene expression levels and experimental conditions while accounting for possible confounding factors, we fit a linear model for each gene using the lmFit function from the limma package (version 3.52.4). Subsequently, we employed the contrasts.fit function from the limma package (version 3.52.4) to compute estimated coefficients and standard errors for specific comparisons among the experimental groups. Finally, to identify differentially expressed genes (DEGs), we conducted differential expression analysis using Empirical Bayes Statistics by applying the eBayes function from the limma package (version 3.52.4)^75,76^.

### GO term enrichment and KEGG pathway analysis

GO term analysis for “biological process” for parasitic coding genes was performed using the PlasmoDB GO term pipeline default settings with *p* ≤ 0.05^74^. KEGG pathway analysis of parasitic genes was similarly performed in PlasmoDB with default settings and *p* ≤ 0.05.

### Statistical analysis

All statistical analyses were performed using GraphPad Prism 9 (GraphPad Software Inc.). For comparison between different treatment groups, two-way ANOVA was performed in combination with Dunnett’s multiple comparison test. Repeated measures (RM) two-way ANOVA with Geisser-Greenhouse correction in combination with Dunnett’s multiple comparison test was applied for comparing different treatment groups over time. A priori assumption of *p* ≤ 0.05 was used for statistical significance. Levels of significance were denoted as *, **, *** in respect to *p* ≤ 0.05, *p* ≤ 0.01 and *p* ≤ 0.001.

### Data presentation

Graphical illustrations were generated with BioRender. Bar and pie charts were plotted with GraphPad Prism 9. All other plots were generated with R^77,78^.

### Supplementary Information

Supplementary Information.

Supplementary Figures.

## Data Availability

The sequencing datasets generated and analyzed during the current study are available in the SRA repository under the accession number PRJNA1001644. All other datasets generated during and/or analyzed during the current study are available from the corresponding author upon reasonable request.
