# Supplementary material for: Starvation induces changes in abundance and small RNA cargo of extracellular vesicles released from Plasmodium falciparum infected red blood cells
Source: Sci Rep. 2023 Oct 27;13:18423. doi: 10.1038/s41598-023-45590-6 (PMC10611735; doi:10.1038/s41598-023-45590-6)

## SUPPLEMENTARY MATERIALS FOR

**Title:**

Starvation induces changes in abundance and small RNA cargo of extracellular vesicles released from *Plasmodium falciparum* infected red blood cells


**Authors:**

Leonie Vetter[1,†] , Amanj Bajalan[1,†], Mohammad Tanvir Ahamed[2], Caterina Scasso[1], Sulman Shafeeq[1], Björn Andersson[3], Ulf Ribacke[1,4*]

**Author affiliations:**

[1] Department of Microbiology, Tumor and Cell Biology, Karolinska Institutet; Solnavägen 9, SE-17165 Solna, Sweden.

[2] Department of Learning, Informatics, Management and Ethics, Karolinska Institutet; Tomtebodavägen 18, SE-17177 Solna, Sweden.

[3] Department of Cell and Molecular Biology, Karolinska Institutet; Solnavägen 9, SE-17165 Solna, Sweden.

[4]Department of Cell and Molecular Biology, Uppsala University; Husargatan 3, SE-75237 Uppsala, Sweden

*Correspondence to: ulf.ribacke@ki.se

† These authors contributed equally to this work


**This PDF file includes:**

Figs. S1 to S7

**Other Supplementary Materials for this manuscript include the following:**

Data file S1

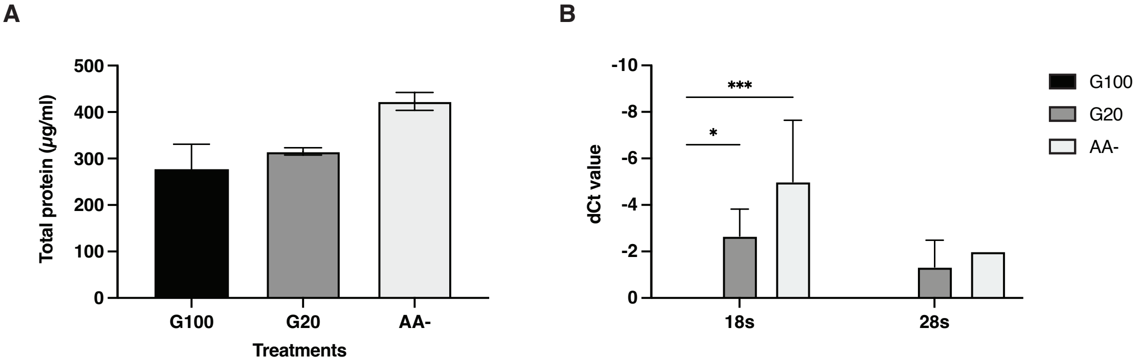


**Supplementary figure S1. Levels of total protein and select parasite transcripts confirm increased release of EVs from starved parasite cultures. (A, B)** Parasite cultures were exposed to different growth conditions (G100, G20 and AA-) for 6h prior to harvesting and purification of EVs from culture supernatants. **(A)** Total protein concentrations in balanced volumes of EV preparations from differently treated cultures quantified by Bradford assays. Results are shown as averages from two biological replicates with error bars representing the range. **(B)** Quantification of *P. falciparum* 18s and 28s ribosomal RNAs of EV preparations from differently treated parasite cultures. Identical volumes of RNA were used as input for RT-qPCR and relative levels of transcripts were computed as delta Ct values ($Ct_{G20/AA-}$ - $Ct_{G100}$). Results are displayed as average delta Ct values from three biological replicates (each assayed in technical triplicates) with error bars representing standard deviations. Asterisks indicate p-values as determined by two-way ANOVA in combination with Dunnet's multiple comparison test (*$p \leq 0.05$, ***$p \leq 0.001$).

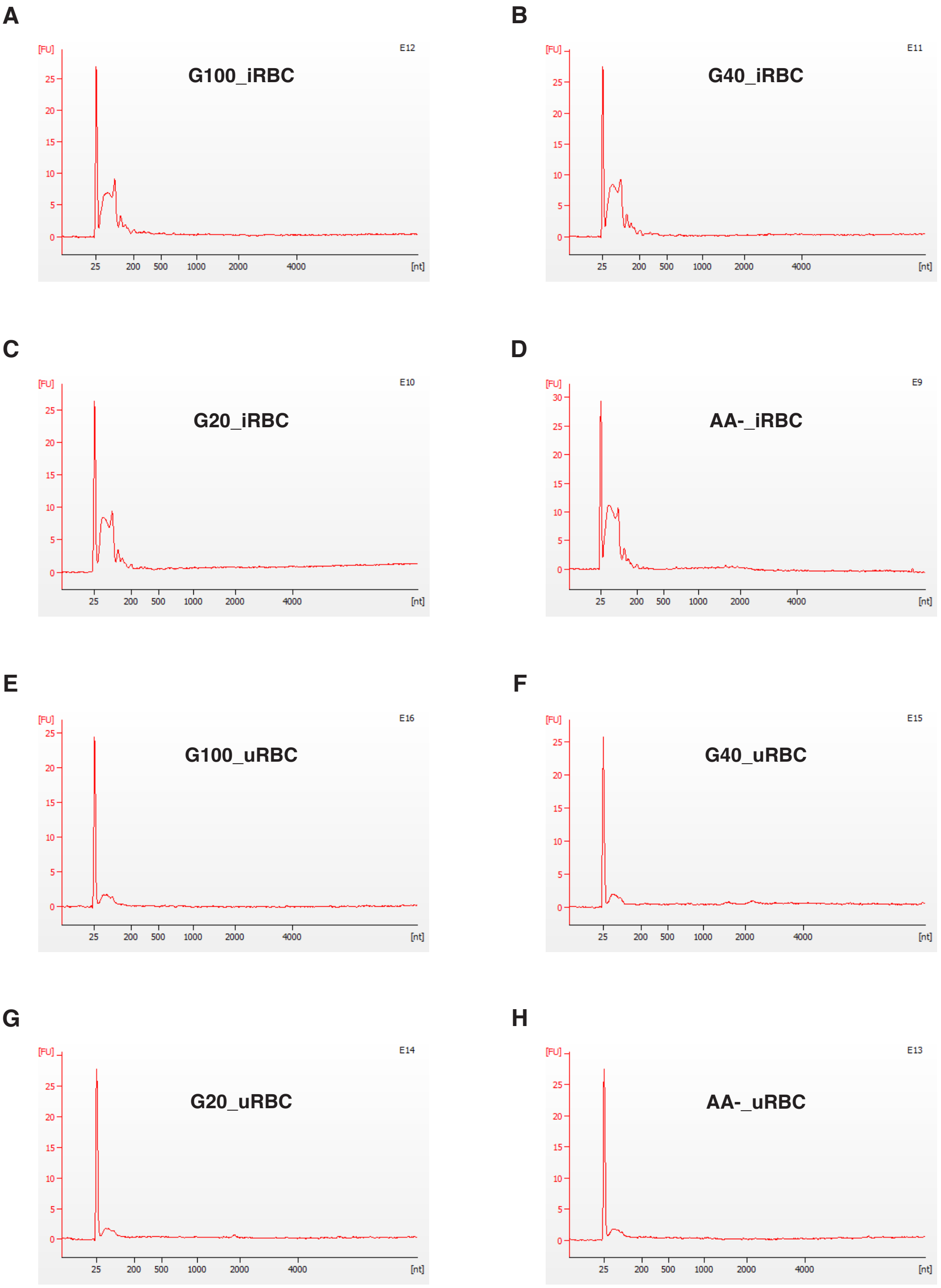


**Supplementary figure S2. The transcriptome of EVs consists of small RNAs and is increased upon parasite infection of RBCs. (A-H)** Total RNA profiles of EVs were determined for preparations from both infected and uninfected RBC cultures with and without alterations to the culture conditions for 4h. One representative Bioanalyzer electropherogram is shown for EVs purified from ring-stage parasite cultures grown **(A)** under normal conditions (G100_iRBC), **(B)** with 40% of normal glucose (G40_iRBC), **(C)** with 20% of normal glucose (G20_iRBC), **(D)** without amino acids (AA-_iRBC) and uninfected RBCs cultured **(E)** under normal conditions (G100_uRBC), **(F)** with 40% of normal glucose (G40_uRBC), **(G)** with 20%

of normal glucose (G20_uRBC) and **(H)** without amino acids (AA-_uRBC). All samples were balanced in terms of number of cells treated and volumes of RNA used as input for Bioanalyzer measurements. The absolute majority of transcripts were smaller than 200 nucleotides in all samples and transcript abundance was greatly elevated in parasite cultures, signifying an increased release of EVs from iRBCs compared to uRBCs.

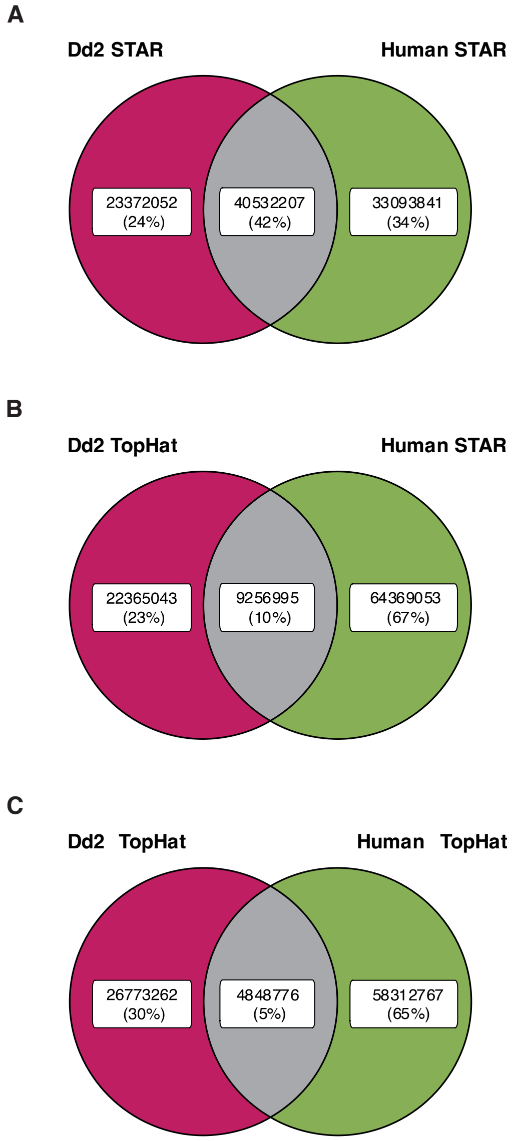


**Supplementary figure S3. Different alignment strategies and their impact on mapping and discrimination of human and parasitic RNA-seq reads. (A-C)** Venn diagrams displaying numbers of mapped reads using different alignment strategies. The fraction of specifically mapped reads to the parasite and human genomes are colored in pink and green respectively while non-specific reads are in grey. **(A)** Reads were mapped against both genomes using STAR, **(B)** against the Dd2 parasite genome using TopHat and against the human genome using STAR and **(C)** against both genomes using TopHat. The latter resulted in less unspecific alignments and was therefore chosen as the strategy of choice for downstream analyses.

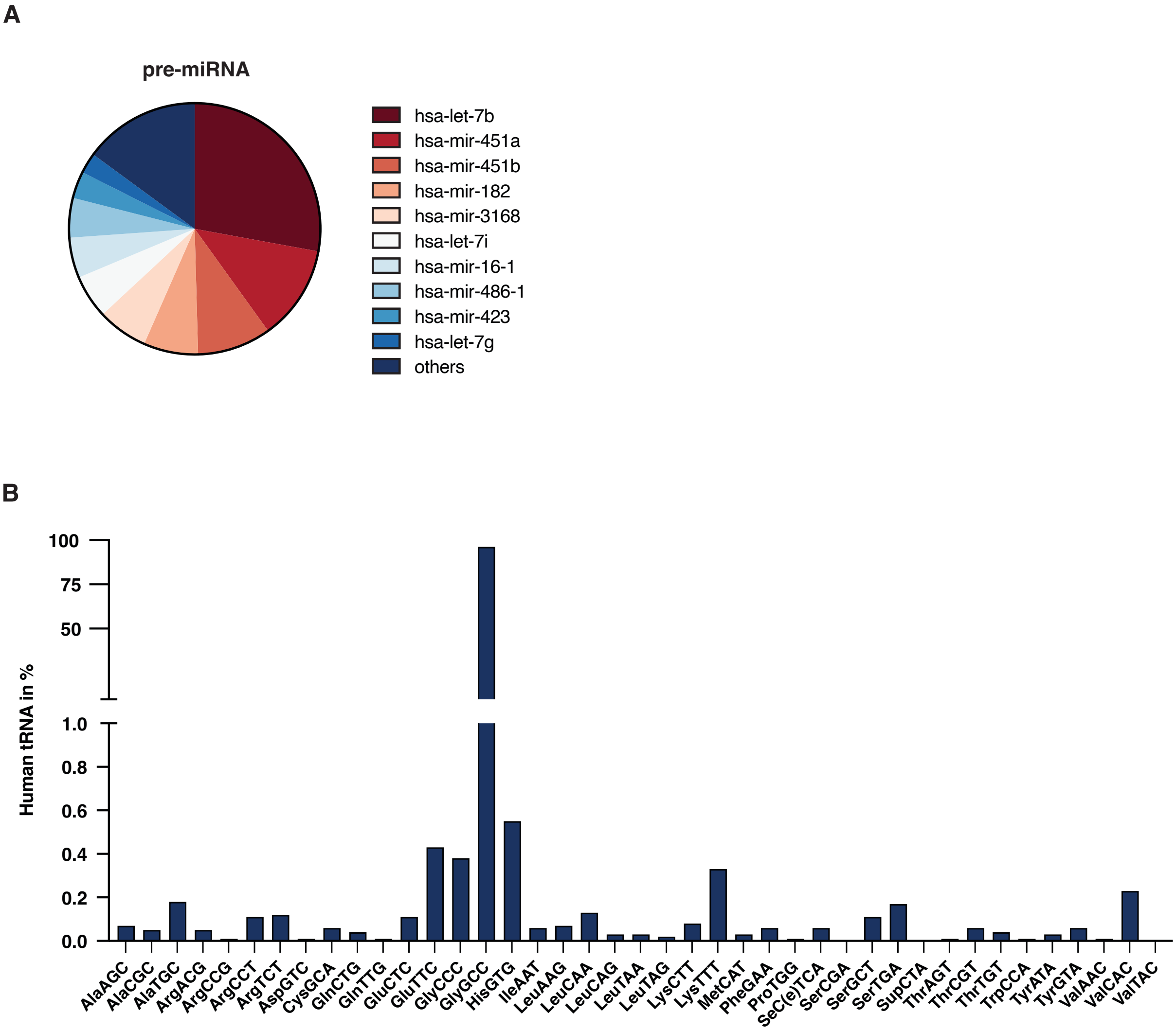


**Supplementary figure S4. The overall distribution of human pre-miRNAs and tRNAs in EVs from parasite cultures. (A)** Pie chart depicting the relative distribution of the most abundant pre-miRNAs found in EVs. **(B)** Bar graphs showing the relative distribution of human tRNA fragments in sequenced EVs, highlighting the dominance of $Gly^{GCC}$.

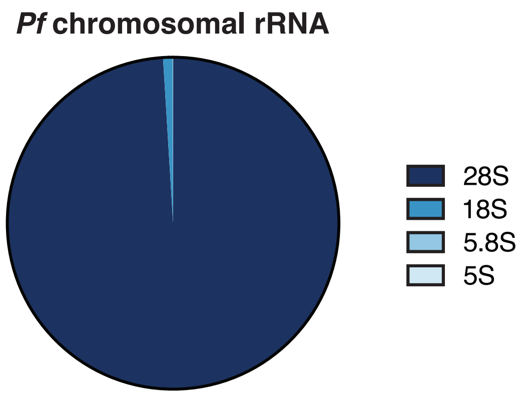


**Supplementary figure S5. The overall distribution of chromosomal rRNA reads in EVs from parasite cultures. (A)** Pie chart depicting the relative distribution of the chromosomal rRNA reads found in EVs, highlighting the dominance of 28S rRNA.

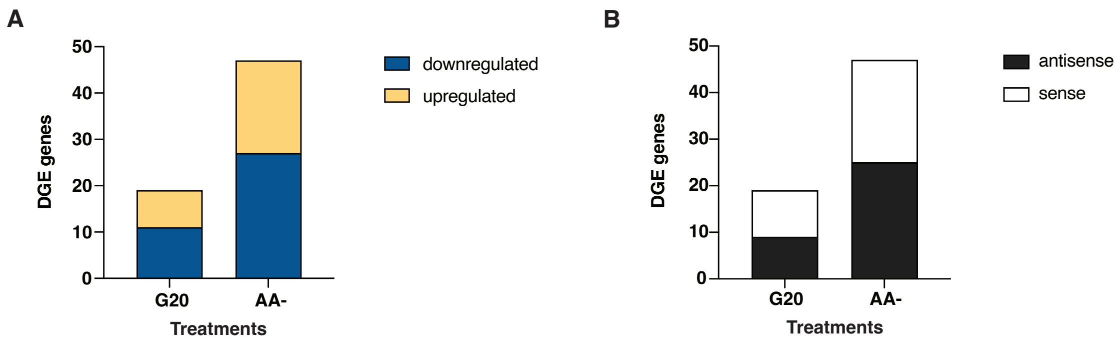


**Supplementary figure S6. Uniform distributions of up- and downregulated transcripts and sense and antisense transcripts among significantly differentially expressed genes. (A)** Bar chart displaying numbers of transcripts found up- or downregulated in EVs from G20 and AA- treated parasite cultures. **(B)** Bar chart depicting numbers of differentially expressed sense and antisense transcripts in EVs from G20 and AA- treated parasite cultures.

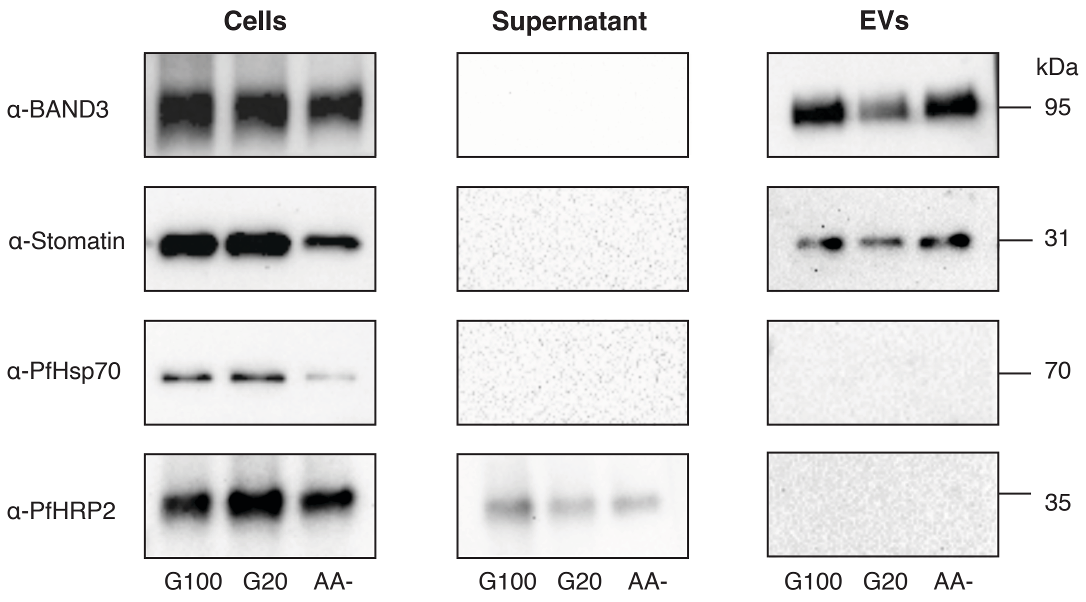


**Supplementary figure S7. Western blot analyses of EV purity.** Protein samples were collected during EV purifications to ensure adequate purity of the preparations. The samples were from ring-stage parasite cultures grown under different conditions (G100, G20 and AA-). Purified EVs, their respective iRBCs and the remaining culture supernatant were investigated. SDS-PAGE separated proteins were analyzed for their presence of known RBC and EV resident human markers using antibodies against Band3 and Stomatin. Similarly, antibodies were used to deduce the presence of parasite proteins known to be either intracellular (PfHsp70) and/or exported outside of the iRBC (PfHRP2). Collectively, the EV preparations were shown to contain proteins known to be EV resident while being devoid of proteins known to be either parasite internal or exported from iRBCs in soluble form.

# Full blot images corresponding to Supplementary figure S7

## Cells

## Supernatant

## EVs

### BAND3

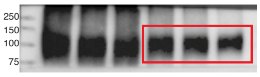


### BAND3

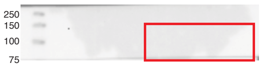


### BAND3

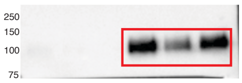


### Stomatin

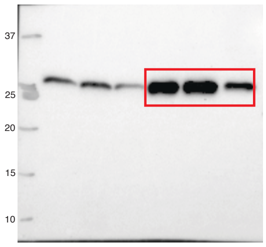


### Stomatin

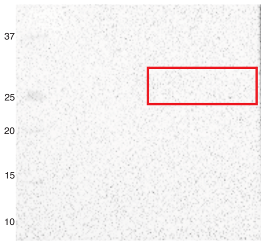


### Stomatin

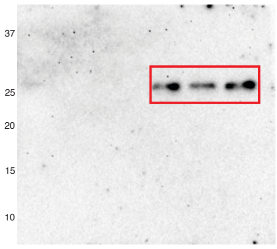


### PfHsp70

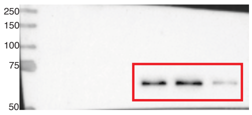


### PfHsp70

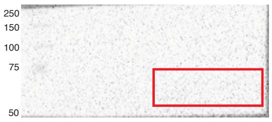


### PfHsp70

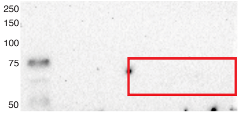


### PfHRP2

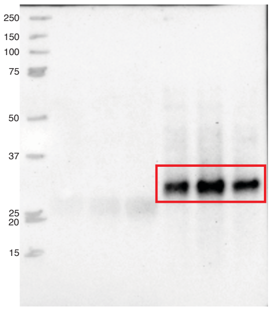


### PfHRP2

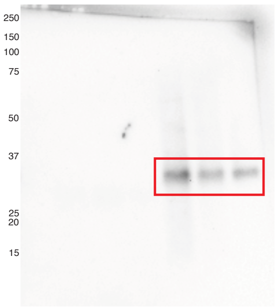


### PfHRP2

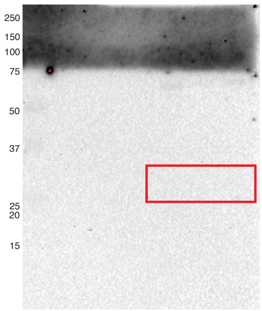

Supplement: Supplementary file 2 — Supplementary Figures. [file 41598_2023_45590_MOESM2_ESM.pdf]
